# Moringa oleifera Supplementation for Reducing Heavy Metal Toxicity and Oxidative Stress in Pregnant Women: Protocol for a Nonrandomized Trial Study

**DOI:** 10.2196/73201

**Published:** 2025-10-31

**Authors:** Hasnawati Amqam, Anna Khuzaimah, Ulfah Najamuddin, Mardiana Ahmad, Muflihatul Muniroh, Ancah Caesarina Novi Marchianti, Saliza Mohd Elias

**Affiliations:** 1 Department of Environmental Health, Faculty of Public Health, Hasanuddin University, Perintis Kemerdekaan KM. 10. Tamalanrea, Makassar, 90245, Indonesia, 62 08114630476; 2 Department of Nutrition, Faculty of Public Health, Hasanuddin University, Makassar, Indonesia; 3 Department of Midwifery, School of Postgraduate, Hasanuddin University, Makassar, Indonesia; 4 Department of Physiology, Faculty of Medicine, Diponegoro University, Semarang, Indonesia; 5 Department of Public Health, Faculty of Medicine, Universitas Jember, Jember, Indonesia; 6 Department of Environmental and Occupational Health, Medical and Sains Faculty, Universiti Putra Malaysia, Seri Kembangan, Malaysia

**Keywords:** antioxidants, environmental exposure, micronutrients, maternal health, detoxification, DNA damage

## Abstract

**Background:**

Heavy metals present in the environment, including lead, cadmium, and mercury, pose significant health risks to pregnant women and fetal development through food, water, and air contamination. Exposure to these metals has been linked to miscarriage, low birth weight (LBW), preterm birth, and developmental issues in children. The mechanism of oxidative stress, characterized by increased 8-hydroxy-2’-deoxyguanosine (8-OHdG) and malondialdehyde (MDA) levels, contributes to DNA damage, genomic instability, and adverse pregnancy outcomes. Additionally, DNA methylation changes induced by metal exposure may further exacerbate these risks. Certain micronutrients play a crucial role in heavy metal detoxification, and *Moringa oleifera*, a locally available plant rich in antioxidants and chelating compounds**,** has demonstrated protective effects against mercury (Hg), lead (Pb), and cadmium (Cd) toxicity in experimental studies. However, intervention studies on pregnant women remain scarce.

**Objective:**

The objective of this study was to evaluate the effect of *M. oleifera* supplementation in reducing heavy metal toxicity and oxidative stress biomarkers, 8-OHdG and MDA, in pregnant women exposed to high levels of heavy metals.

**Methods:**

A quasi-experimental, nonrandomized pre-post test design is used. Pregnant women with elevated heavy metal levels, identified through initial screening, will be included in the intervention group, receiving *M. oleifera* supplementation for 2 months. The control group will consist of women from similar geographical regions who will not receive the intervention. Primary outcomes will include changes in heavy metal concentrations, measured using inductively coupled plasma mass spectrometry (ICP-MS). Secondary outcomes will focus on reductions in oxidative stress biomarkers, measured via enzyme-linked immunosorbent assay (ELISA). Statistical analyses, including analysis of covariance (ANCOVA), will be used to adjust for baseline differences between the groups.

**Results:**

A total of 26 mothers for each group have participated. As of February 2025, the laboratory analyses have been ongoing, and the result is expected to be published at the end of 2025. The protocol anticipates that the intervention group will show a significant reduction in both heavy metal levels and oxidative stress biomarkers compared to the control group, suggesting the potential efficacy of *M. oleifera* in detoxifying heavy metals and reducing oxidative stress.

**Conclusions:**

This study is expected to provide preliminary evidence on the potential effectiveness and safety of *M. oleifera* supplementation for reducing heavy metal toxicity and oxidative stress in pregnant women.

## Introduction

Heavy metals such as cadmium (Cd), mercury (Hg), and lead (Pb) exist in water, air, and food, contributing to cumulative exposure in humans [1,2]. According to the World Health Organization (WHO), Pb exposure alone is responsible for nearly 1 million deaths annually and more than 21 million disability-adjusted life years (DALYs) lost, with more than 95% of this burden occurring in low- and middle-income countries (LMICs) [3]. Prenatal exposure to Pb and other heavy metals is particularly harmful as it has been linked to miscarriage, preterm birth, low birth weight (LBW), and developmental problems in children [4-6]. Studies have shown that no level of lead exposure is considered safe during pregnancy, and even low-dose exposures can disrupt fetal development. These risks highlight the urgent need for public health interventions targeting environmental metal exposures in vulnerable populations, especially among pregnant women in LMICs where monitoring and remediation remain limited.

In Makassar, Indonesia, 717, 347, and 816 LBW cases were reported in 2021, 2022, and 2023, respectively. Preliminary investigation also revealed significantly elevated levels of blood Pb (1.35–101.00 μg/L) and Cd (0.55–17.00 μg/L), exceeding reference values in various countries [7]. For context, the US Centers for Disease Control and Prevention (CDC) considers blood Pb levels above 5 μg/dL (50 μg/L) as elevated in adults [8-10], while the WHO identifies Cd concentrations exceeding 1 μg/L in blood as potentially toxic. These findings highlight a concerning level of environmental exposure during pregnancy. Although specific to Makassar, such elevated exposures reflect broader environmental health challenges in urban Indonesia and Southeast Asia, where industrial emissions, traffic pollution, and inadequate regulation contribute to rising heavy metal contamination.

One key mechanism underlying the heavy metal toxicity is oxidative stress, which causes damage to lipids, proteins, and DNA. Biomarkers such as malondialdehyde (MDA) and 8-hydroxy-2’-deoxyguanosine (8-OHdG) reflect oxidative damage and are commonly elevated in metal-exposed individuals  [7-9]. Additionally, heavy metals can disrupt DNA methylation [10], an epigenetic process critical for normal fetal development, thereby contributing to adverse pregnancy outcomes  [11-13]. Heavy metals such as Pb, Cd, and Hg exert toxic effects partly through the generation of reactive oxygen species (ROS), leading to oxidative stress [14]. This imbalance between ROS and antioxidant defenses causes damage to cellular lipids, proteins, and DNA, as reflected by increased levels of oxidative stress biomarkers such as 8-OHdG and MDA [15,16]. These molecular injuries are particularly harmful during pregnancy as they disrupt placental development and fetal growth processes [17,18]. Studies have shown that oxidative stress plays a central role in the pathogenesis of adverse pregnancy outcomes [19], including preeclampsia [20], miscarriage [21], LBW [22], and preterm birth [23]. A previous study by Zinia et al [24] indicated that high maternal blood Cd levels (0.62 μg/L in early pregnancy and 0.70 μg/L in late pregnancy), Pb levels (early pregnancy 0.74 vs late pregnancy 0.70 μg/dL), and Hg levels (early pregnancy 2.37 vs late pregnancy 1.95 μg/L) were inversely associated with birth weight.

Although certain micronutrients such as zinc and cysteine may assist in the detoxification of heavy metals [25], their use as targeted interventions remains inconsistent and often inaccessible to at-risk populations. Therefore, *Moringa oleifera* has emerged as a promising, locally available resource. Moringa contains flavonoids, phenolic acids, glucosinolates, and phytates and exhibits antioxidant and metal-chelating properties that can mitigate the toxic effects of heavy metals [26,27]. These compounds have been shown in in vitro and in vivo studies to counteract oxidative stress and inflammation induced by toxicants, including heavy metals.

Beyond laboratory studies, there is growing evidence of moringa’s nutritional and therapeutic effects in humans. Moringa leaf supplementation has shown improvements in hemoglobin levels in children with iron deficiency anemia and increased breast milk production in postpartum women [28]. In a clinical trial, moringa leaf powder (0.5 g/d) led to significant reductions in body weight, blood pressure, and lipid profiles in overweight individuals with hyperlipidemia [29]. In pregnant women, moringa has emerged as a beneficial supplement, particularly in enhancing maternal and neonatal health. A study in southern Ethiopia found that the consumption of moringa was associated with an increase in birth weight by approximately 115.77 g, suggesting its potential role in improving neonatal outcomes [30]. Moringa leaves are rich in iron, which is crucial for preventing anemia during pregnancy. A study in Karachi reported that pregnant women consuming moringa leaf powder showed hemoglobin levels of 9.09 g/dL in the third trimester, indicating a potential benefit in managing anemia [31]. No contraindications for moringa use during pregnancy were reported in the reviewed studies, suggesting it is generally safe for consumption [32].

Although moringa grows abundantly in many regions of Indonesia, particularly in Makassar, it is typically consumed sporadically as a vegetable, with unmeasured doses and limited awareness of its therapeutic potential. In other areas, such as Java, consumption is even more limited. These factors underscore the need for a standardized, supplement-based intervention to realize public health benefits of moringa. Despite the well-documented risks of heavy metal exposure during pregnancy, interventional studies remain scarce, particularly in Indonesia. To date, there has been no clinical trial examining the efficacy of moringa in the detoxification of heavy metals among pregnant women. In addition, most existing research is observational and lacks an evaluation of strategies to reduce maternal metal levels or oxidative damage. This study seeks to address this gap by investigating the effect of standardized *M. oleifera* supplementation on heavy metal level and oxidative stress reduction in pregnant women. We hypothesize that pregnant women receiving *M. oleifera* supplementation will have significantly lower blood heavy metal concentrations and oxidative stress biomarkers than before receiving.

## Methods

### Study Design

This study is an ongoing quasi-experimental investigation that uses a nonrandomized pre-post test methodology. Expectant mothers were divided into 2 cohorts, one receiving an intervention and the other serving as a control. This study applied a nonrandomized pre-post test design due to practical considerations related to (1) practical management, (2) geographical differences, and (3) time constraints.

First, practical management considers that managing the study is easier when specific locations are designated for the intervention group and other locations for the control group. This approach ensures that the intervention (*M. oleifera* supplementation) can be efficiently delivered and monitored in each location without the complexity of randomizing participants across multiple sites. Furthermore, grouping participants by location helps streamline logistical operations such as monitoring compliance and ensuring consistent follow-up over the course of the intervention period.

Second, although the geographical settings of the intervention and control groups may differ, they were comparable in terms of demographic characteristics and environmental conditions. This allowed us to evaluate the effects of moringa supplementation in different geographical settings while maintaining comparability between groups. In addition, by focusing on distinct locations for intervention and control, we ensure better resource allocation and minimize the logistical challenges that would arise from attempting to randomize participants across a broader region.

Third, time was considered a constraint due to limitation in the laboratory. We could not receive heavy metal analysis results from the initial screening at the same time for all participants. Therefore, women with high levels of heavy metals at the first screening were included in the intervention group to avoid delays. This ensures that these participants can begin the 2-month intervention with *M. oleifera* as soon as possible after obtaining the result, considering the limited research timeframe. Furthermore, because we limited the overall time for the study, randomizing participants could cause delays in implementing the intervention and complicate the process of ensuring that those with high levels of heavy metals receive timely treatment.

A nonrandomized approach allows for a more timely intervention, while still enabling an effective comparison between the intervention and control groups. Statistical methods will be used to control for baseline disparities and account for potential differences between the groups.

### Moringa Leaf Extract Supplementation

#### Collecting Moringa Leaves

The moringa leaves used in this study were collected from moringa trees growing in the Gowa and Takalar regencies. Fresh and healthy leaves were used for supplementation. The leaves chosen were mature, dark green leaves from the tenth stalk down from the tip of the branches. A previous study [33] has shown that mature moringa leaves exhibit a higher antioxidant activity than younger leaves.

#### Washing and Drying

The harvested moringa leaves were washed by dipping them in water and rinsing them several times with running water to remove any dirt or impurities. After washing, the leaves were air-dried for 2 h to remove the excess water. The leaves were separated from the stalks. The drying process was conducted out in an oven at a temperature of 30‐40 °C for 3‐4 hours or until the leaves reached a moisture content of less than 10%.

#### Extraction

The dried moringa leaves were ground into a fine powder using mesh 60. The powder was extracted using distilled water (Aquadest). The extraction process involved soaking moringa powder in distilled water, after which the mixture was squeezed and filtered through a muslin cloth to separate the extract from the residue. The obtained extract was further processed using a rotary evaporator and dried using a freeze dryer until a dry extract was produced.

#### Capsule Supplement Production

The dry extract was ground again using mesh 100 to obtain a fine extract powder. This powder was then mixed with corn starch as a filler in a 4:1 ratio (400 mg of moringa extract and 100 mg of corn starch per capsule). Each capsule contained 400 mg of moringa leaf extract. Participants in the intervention group received 2 capsules twice daily (totaling 2000 mg/d) for a period of 8 weeks, beginning immediately upon enrollment (between gestational weeks 20‐32). In cases where participants gave birth before completing the 2-month supplementation period, they were instructed to continue consuming the remaining *M. oleifera* supplements until the full course was completed. This approach aimed to ensure consistency in the intervention dosage, regardless of the timing of delivery, and to maintain the study’s internal validity.

#### Quality Control

After production, supplementation with moringa leaf extract capsules was tested to ensure weight uniformity and quality, including checks for moisture content, microbial presence, and organoleptic tests to ensure product safety and quality. The dosage of moringa leaf supplementation in this study was determined based on several previous safety studies [34-36], which indicated that the consumption of moringa leaves is safe at doses up to 2000 mg/day for humans and has the potential to be a safe source of antioxidants for pregnant women.

### Participant Recruitment

This research has been conducted in Makassar City, specifically at 6 community health centers where expectant women receive prenatal services. The 6 selected community health centers are located in areas with varying environmental characteristics, including industrial zones, regions near landfill sites, and coastal areas. While the types of pollution sources differ, all sites share a common feature: exposure to potential environmental contaminants. Each represents an urban setting with a plausible risk of heavy metal exposure through air, water, and food pathways. Thus, despite differences in specific pollution types, the study sites are considered comparable in terms of overall environmental risk exposure. This selection strategy ensures that the intervention and control groups are drawn from communities facing similar environmental health challenges. Moreover, such variation may enhance the generalizability of findings across different urban pollution contexts. Three centers were assigned as intervention sites and 3 as control sites based on operational convenience and to avoid information spillover between groups.

The study will include 59 pregnant women in each group of intervention and control who underwent an examination at health centers in Makassar and fulfilled the following inclusion criteria: (1) pregnancy during the second or third trimester, (2) intention to deliver in Makassar, (3) living in Makassar for a period exceeding 1 year, and (4) blood levels of Hg, Pb, or Cd exceeding the reference value. Participants will be excluded based on self-report during interview if they (1) are having twin pregnancy, (2) had known chronic medical conditions, such as diabetes, cancer, kidney diseases, (3) are taking other antioxidant or mineral supplements beyond standard care, (4) are diagnosed with micronutrient deficiencies, (5) relocate during the intervention period, and (6) are unwilling to comply with follow-up procedures.

The required sample size was calculated using an estimated effect size (Cohen *d*) of 0.42, derived from a previous intervention study in South Sulawesi, which assessed the effects of *M. oleifera* supplementation on oxidative stress biomarkers in pregnant women exposed to passive smoking [37]. The study reported a mean difference in 8-OHdG levels between treatment groups of approximately 12.96 ng/mL with a pooled SD of ~30.68 ng/mL, yielding a moderate effect size. Using a 1-sided test with *α*=.05 and 80% power, the minimum required sample size is 37 participants per group. To account for an anticipated 20% dropout rate, the sample size was increased to 44 participants per group, yielding a total of 88 participants.

Approximately 200 pregnant women attending routine antenatal care (ANC) visits at the community health centers were sequentially approached and invited to participate in the study. They were first interviewed to determine eligibility based on inclusion and exclusion criteria. Those meeting the criteria were provided with detailed information about the study and asked to provide written informed consent. Following consent, a blood sample was collected to assess baseline levels of heavy metals.

Only participants with elevated levels above reference thresholds were considered eligible for inclusion in the intervention phase. For the intervention group, eligible participants were contacted and provided with *M. oleifera* supplements along with instructions for daily consumption over a 2-month period. They were informed that a follow-up blood collection would occur at the end of the supplementation period during a routine visit.

For comparison, the control group was recruited from a different health center in a separate subdistrict administrative unit that did not receive moringa supplementation. This approach minimized contamination between groups and allowed for feasible implementation of the intervention. Follow-up blood sampling was also conducted after 2 months, aligned with their next scheduled antenatal visit, but without providing moringa supplementation.

### Ethical Considerations

This study was approved by the Ethics Committee of the Public Health Faculty (number: 1549/UN4.14.1/TP.01.02/2024). All participants were informed about the study’s purpose, procedures, potential risks, and right to withdraw at any time before data collection. Written informed consent was obtained after that. Initial screening for heavy metal exposure was conducted after informed consent was obtained. All historically collected data will be deidentified before the analyses. The participants received the equivalent of around US $6.25 for compensation.

### Research Procedure

#### Overview

**Figure 1.**
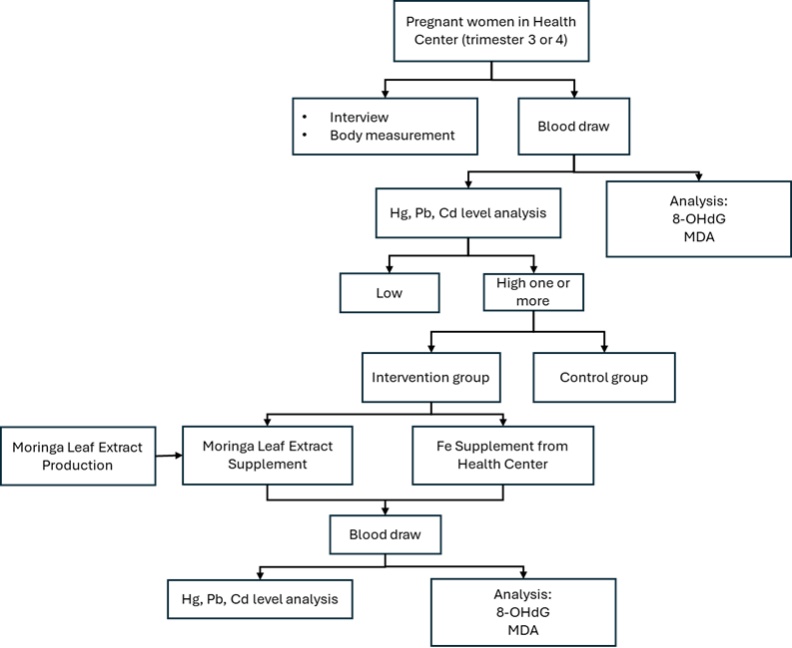
Research step. Fe: ferrum (iron supplement); Hg: mercury; Pb: plumbum (lead); Cd: cadmium; 8-OHdG: 8-hydroxy-2'-deoxyguanosine; MDA: malondialdehyde.

The research step is presented in Figure 1.

#### Data Collection

An interview has been conducted with pregnant women using a questionnaire that contained questions about their characteristics and potential exposure sources. Data on food/supplement intake will also be obtained using a 24-hour recall form and a Food Frequency Questionnaire (FFQ) to assess exposure to heavy metals through food. Each participant provided 10 mL of venous blood sample, acquired using a tube lined with ethylenediaminetetraacetic acid (EDTA) and kept at a temperature of 2‐8 °C until analysis. For MDA and 8-OHdG analyses, blood samples were centrifuged to obtain plasma and stored at temperatures of −80 °C until analysis.

Heavy metals, 8-OHdG, and MDA concentrations as the outcomes will be measured at 2 time points: baseline (preintervention) and postintervention (after 8 weeks). The measurements at baseline will be performed at enrollment, before the administration of *M. oleifera* supplementation. The measurements at postintervention (after 8 weeks) will be performed at the end of the 2-month supplementation period, when the same biomarkers (blood heavy metals, 8-OHdG, MDA) will be reassessed to evaluate changes.

### Intervention Group

In addition to receiving iron capsules, which are standard supplements provided by the health centers as part of the national prenatal care program, participants in the intervention group also received *M. oleifera* extract supplementation at a dose of 2000 mg/day, administered as 2 capsules taken twice daily (totaling 2000 mg/day) for a continuous period of 8 weeks. Supplementation began immediately upon enrollment, which occurred between gestational weeks 20 and 32. Therefore, the regimen lasted from the time of enrollment until 8 weeks later, typically ending between weeks 28 and 40 of gestation. For participants enrolled in the late third trimester who delivered before completing the full 8 weeks, they were instructed to continue consuming the remaining *M. oleifera* supplements until the full course was completed. This approach aimed to ensure consistency in the intervention dosage, regardless of the timing of delivery, and to maintain the study’s internal validity.

To support adherence, we collaborated with health cadres at each participating health center. The cadres will be responsible for supervising, reminding, and ensuring that the mothers consistently take the moringa capsules. A WhatsApp group was established specifically for the cadres to facilitate regular communication, provide reminders, and monitor the supervision process throughout the 8-week supplementation period. We have also added a simple adherence log that will be maintained by the cadres, documenting capsule intake daily based on direct questioning or observation. These dual approaches—cadre monitoring and pill counts—will allow us to triangulate adherence data and better distinguish true intervention effects from variability due to compliance.

Participants will be actively monitored for adverse events throughout the 8-week intervention period. At the follow-up visit, participants will be asked about any symptoms or side effects experienced, using a structured checklist and open-ended questions. Specific adverse events of interest include gastrointestinal discomfort (nausea, vomiting, and diarrhea), allergic reactions (skin rash and swelling), and general intolerance to the supplement.

Any serious adverse events, including pregnancy complications such as preeclampsia, gestational diabetes, preterm labor, or hospitalizations, will be recorded and reviewed by the study physician. Although these are not primary outcomes, maternal and fetal complications will be documented to ensure a complete safety profile.

Prior evidence suggests that *M. oleifera* supplementation at a dose of 2000 mg/day is safe for human use, including during pregnancy. However, all safety data will be recorded in participant case report forms, monitored by the study team, and summarized in the final report.

### Control Group

Mothers in the control group will receive only the standard iron supplement. Although iron itself may influence oxidative stress, this effect can be controlled by equalizing iron intake across groups. They will be counseled on dietary sources of antioxidants and offered *M. oleifera* supplementation after study completion if desired. This delayed intervention strategy has been approved by the local ethics committee and was explained during informed consent.

To minimize the risk of measurement and performance bias, participants in the control group were not informed about the specific intervention given to the other group.

### Biomarker Analysis

#### Heavy Metal Analysis

The measurement was conducted to determine Hg, Pb, and Cd levels in the blood of pregnant women during the second or third trimester. Heavy metals were analyzed using inductively coupled plasma mass spectrometry (ICP-MS) (Thermo Fisher Scientific, Bremen, Germany) and performed in micrograms per liter. To ensure analytical accuracy and method validation, we used certified reference materials (CRM BCR®-636, lyophilized human blood, Joint Research Center-JRC, European Commission) as a quality control standard. For quality assurance and quality control (QA/QC), calibration curves were generated using multielement standard solutions of known concentrations. Blank samples were included in each analytical batch to monitor contamination.

#### Oxidative Stress Analysis

In this study, 8 OHdG and MDA are used as biomarkers of oxidative stress because they capture two complementary pathways: DNA oxidative damage and lipid peroxidation, respectively. Both markers have been validated in pregnant populations exposed to heavy metals such as Pb, Cd, and Hg. Alternative enzymatic antioxidants (eg, glutathione peroxidase, catalase) require immediate enzyme activity preservation, which cannot be guaranteed under our field conditions; 8 OHdG and MDA, by contrast, remain stable in frozen plasma and are therefore better suited to the study’s logistics.

Plasma concentrations of MDA (nmol mL⁻¹) will be quantified in duplicate using a commercially available ELISA kit (Thermo Fisher Scientific, Bremen, Germany). 8 OHdG (ng mL⁻¹) will be measured in duplicate with a validated ELISA kit (Bioassay Technology Lab, Shanghai Korain Biotech Co. Ltd). For each assay run, an 8-point standard curve, reagent blanks, and low-/high-quality control samples will be included. Concentrations will be derived from the standard curves using a 4-parameter logistic model, and any sample with a coefficient of variation > 10 % on duplicate readings will be reassayed. Laboratory technicians will be blinded to group allocation to minimize measurement bias.

### Data Analysis

All analyses will be conducted using the R statistical software (latest version). Data entry and cleaning will be conducted by trained staff using a double-entry verification. For 24-hour food recall data, participants will be asked to estimate portion sizes using visual aids, and data will be reviewed during collection for completeness and clarity. For the FFQ, if participants leave implausible answers (eg, extremely high frequency), the data will be reviewed and clarified during the interview or excluded from analysis after documentation. Implausible intake frequency will be cross-checked with interview notes and food recall summaries to determine plausibility. An intention-to-treat (ITT) analysis will be performed to preserve the benefits of initial group allocation. For missing or partial responses, multiple imputation will be considered for covariates in the adjusted models. For missing outcome data at follow-up, last observation carried forward (LOCF) will be used when feasible. Sensitivity analyses will be conducted to assess the robustness of findings under different missing data assumptions. Outliers will not be automatically excluded unless clear reporting errors are confirmed. Outliers in biomarker measurement will be identified using the interquartile range method and retained unless proven to result from analytic error or sample contamination. All statistical analyses will be conducted with and without outliers to assess the robustness of the results.

Descriptive statistics will summarize participant characteristics. All continuous variables, including heavy metal concentrations and biomarker levels, will be checked for normality before analysis. In the case of non-normal distributions, appropriate data transformations (eg, log transformation) will be applied. If normality cannot be achieved, nonparametric tests (eg, Mann–Whitney *U* or Wilcoxon signed-rank test) will be used as alternatives. Analysis of group differences (pre- and postintervention). For each group, analysis will be conducted using the paired *t* test or Wilcoxon signed-rank test, depending on data distribution, to assess changes in heavy metals, MDA, and 8-OHdG levels before and after intervention. The significance level (alpha) will be set at .05, and all tests will be one-tailed.

For comparison between the groups (intervention vs control), analysis will be conducted using the difference-in-differences (DiD) method to measure the difference in changes between the intervention and control groups. The results will provide an overview of the impact of moringa leaf supplementation on the reduction of heavy metal levels and prevention of DNA damage in pregnant women. To address potential selection bias and confounding due to the nonrandomized design, we will use analysis of covariance (ANCOVA) to adjust for baseline differences in key variables such as maternal age, gestational age, education level, and baseline levels of heavy metals, MDA, and 8-OHdG.

Assumptions for ANCOVA will be checked using diagnostic plots and tests. Normality of residuals will be checked using the Shapiro–Wilk test and Q-Q plots. The homogeneity of variance test will use the Levene test from the car package. Linearity and homoscedasticity will be checked using residual versus fitted plots.

The interaction effects between intervention status and baseline heavy metal levels will be examined by adding interaction terms to the ANCOVA models. Additionally, stratification will be considered a sensitivity analysis to minimize bias. Baseline heavy metal levels will be included as covariates in the statistical models to account for variation in environmental exposure across recruitment areas. In addition, we will conduct exploratory subgroup analyses to assess potential effect modifiers. Outcomes will be examined separately for each heavy metal to assess whether the reduction effect of *M. oleifera* differs by metal type. Additional exploratory subgroup analyses may include stratification by trimester of pregnancy, nutritional status (BMI), and geographic location/pollution source.

If substantial differences are observed, these findings will be interpreted cautiously due to limited statistical power. All subgroup analyses will be considered hypothesis generating and reported transparently. If no significant interaction is found, primary analyses will focus on aggregate outcomes across all participants. Reduction in each individual heavy metal will be reported separately, along with a composite index. No interim analyses or stopping rules are planned due to the short duration of the trial and the nature of the design.

## Results

Overview

The study was funded in 2024 and data collection began in July 2024. A total of 26 mothers in each group have participated. As of February 2025, the laboratory analysis is ongoing for the second step. We expect the results to be published at the end of 2025.

### Expected Outcomes

#### Reduction in Blood Heavy Metal Levels

The primary outcome of this study is the reduction in blood Pb/Cd/Hg level. The hypothesis is that supplementation with *M. oleifera* will result in a reduction in the blood levels of heavy metals in pregnant women compared to those in the control group. Hence, the expected results are as follows: (1) A measurable decrease in the blood Pb/Cd/Hg concentration will be observed in the group receiving *M. oleifera* supplementation for 2 months. (2) The intervention group is expected to show a greater reduction in blood heavy metal levels than the control group, based on pre- and post-test analyses.

Scientific implications expected are that the result would provide evidence that *M. oleifera* has the potential to detoxify, thereby demonstrating that it is a safe and effective approach to reducing heavy metal accumulation in expectant mothers, who are at a higher risk of exposure to environmental pollutants.

#### Reduction in 8-OHdG and MDA Concentrations

Hypothesis: *M. oleifera* will reduce 8-OHdG and MDA levels in pregnant women compared with the control group.

The expected results are as follows: (1) Significant reduction in 8-OHdG and MDA levels in the intervention group, indicating a protective effect against oxidative stress and DNA damage. (2) The intervention group will show lower levels of 8-OHdG and MDA after the intervention than the control group.

These findings will indicate that the antioxidant compounds of *M. oleifera* are effective in inhibiting DNA damage and protecting cells against oxidative stress. This is especially important for pregnant women to ensure fetal health and reduce complications.

#### Safety and Tolerability of *M. oleifera*

Hypothesis: *M. oleifera* supplementation will be safe for pregnant women, with no significant adverse effects reported during the intervention period. Hence, the expected results are as follows: (1) Minimal to no adverse effects in the group receiving *M. oleifera* supplementation. (2) This safety profile is consistent with previous studies indicating that 2000 mg/day is safe for pregnant women.

This will provide further evidence that *M. oleifera* is a safe supplement for pregnant women, supporting its use in future clinical applications and maternal health programs.

In the final analysis, changes in heavy metal levels (Pb, Cd, and Hg) will be reported individually, as well as in aggregate if appropriate. Similarly, biomarkers of oxidative stress (8-OHdG and MDA) will be analyzed and reported separately. While the study is not powered for definitive subgroup comparisons, exploratory reporting by relevant characteristics (eg, trimester, nutritional status, and baseline exposure level) will be presented to generate hypotheses for future studies.

## Discussion

### Principal Anticipated Findings

This study is expected to demonstrate that *M. oleifera* supplementation during pregnancy reduces blood levels of heavy metals and decreases oxidative stress biomarkers (8-OHdG and MDA). It also aims to confirm the safety and tolerability of moringa in pregnant women, laying the groundwork for its use as a low-cost, locally available intervention for mitigating environmental pollutant-related health risks.

Several animal studies have shown that moringa leaf extracts can significantly reduce heavy metal accumulation in vital organs and mitigate oxidative stress caused by Pb, Cd, and Hg exposure. These effects are attributed to its high content of flavonoids, phenolic acids, glucosinolates, and carotenoids, which act as free radical scavengers and binders of toxic metals.

While direct clinical evidence in pregnant women remains limited, moringa has been used traditionally in many parts of Indonesia and other countries as a nutritional supplement and natural remedy, including during pregnancy. Small-scale human studies in nonpregnant populations have reported improvements in antioxidant status and reductions in lipid peroxidation following moringa supplementation. This study assumes that the biological mechanisms observed in preclinical research—namely, antioxidant protection and chelation of toxic metals—will translate to similar detoxifying effects in pregnant women.

Importantly, while this study does not evaluate direct clinical outcomes (eg, birth weight, gestational age), the primary focus is on biomarker-based improvements, which are commonly used as surrogate indicators of fetal and maternal risk. The findings are expected to strengthen the biological plausibility for moringa as a preventive intervention in polluted environments and inform the design of future clinical outcome studies.

While the study aims to demonstrate reductions in blood heavy metals and oxidative stress biomarkers following *M. oleifera* supplementation, such findings—if observed—should be interpreted with caution. Positive outcomes would indicate that moringa is a promising candidate for reducing biochemical risk factors associated with environmental exposures in pregnancy. However, this study does not assess long-term or clinical outcomes (eg, fetal growth and birth weight) and therefore cannot confirm improvements in maternal or fetal health. Additionally, the study does not evaluate cost-effectiveness or scalability of implementation.

Conversely, if no significant effects are observed, several factors could explain this, including insufficient dosage, short intervention duration, limited sample size, or individual variability in absorption or baseline exposure. These considerations will guide future study design and refinement of the intervention strategy. Ultimately, findings from this study should be viewed as hypothesis-generating, laying the groundwork for larger, randomized trials with extended follow-up and clinical endpoints.

### Comparison to Prior Work

Previous studies have supported the antioxidant potential of *M. oleifera*, particularly its ability to reduce oxidative stress. For instance, in infertile women, moringa supplementation reduced oxidative stress markers and slightly increased antioxidant biomarkers, suggesting potential benefits for reproductive health [38]. Another study [39] demonstrated that supplementation with moringa leaf extract decreased levels of MDA in pregnant women, suggesting its role in reducing lipid peroxidation. This supports the hypothesis of the present study regarding moringa’s capacity to attenuate oxidative stress.

However, while there is growing interest in the detoxifying potential of moringa, evidence of its effect on heavy metal levels in humans remains limited. Currently, most studies demonstrating metal-chelating and detoxification effects of moringa have been conducted in animal models [40-46], showing efficacy in reducing Hg, Pb, and Cd accumulation. These include experimental studies in rodents that found significant reductions in blood and tissue metal concentrations after moringa supplementation.

This study extends this line of research by being one of the first protocol studies designed to evaluate whether these benefits translate into pregnant human populations exposed to environmental heavy metals. By integrating biomarker-based outcomes (heavy metals, 8-OHdG, and MDA), this study aims to address a critical evidence gap in prenatal environmental health interventions.

Thus, if our hypothesis is confirmed, it will add to a growing body of evidence supporting the nutritional role of antioxidant-rich plants in maternal health and offer novel evidence of moringa’s detoxifying role in reducing human heavy metal burden during pregnancy.

### Strengths and Limitations

This study has several limitations that should be acknowledged. First, the nonrandomized design introduces a risk of selection bias and limits internal validity. While the selection of different locations for the intervention and control groups was based on logistical and feasibility considerations, this may lead to differences in participant characteristics and environmental exposures. To mitigate this, statistical adjustments such as ANCOVA will be used, and effort has been made to select sites with comparable demographic profiles.

Second, blinding was not feasible in this trial—neither participants nor investigators are blinded to the intervention. This raises the potential for performance and detection bias, although this risk is minimized by the use of objective laboratory-based biomarkers assessed by independent laboratory technicians.

Third, the sample size may limit statistical power, particularly for detecting small effects or conducting subgroup analyses. As a result, findings from this study should be interpreted as preliminary and exploratory, forming a basis for future hypothesis-driven studies.

Fourth, the short duration of intervention (2 mo) and the use of biomarkers instead of clinical outcomes (such as birth weight or gestational age) limit the ability to draw conclusions about long-term maternal or fetal health impacts. While changes in oxidative stress and metal levels are valuable surrogate outcomes, future studies should evaluate direct clinical endpoints.

Finally, although both study groups were drawn from similar urban settings, environmental variability (eg, differences in pollution sources or background exposure levels) may confound the results. These residual environmental factors could influence outcomes independently of the intervention and are difficult to fully control.

Despite these limitations, the study addresses an important gap by testing a locally available, low-cost, and traditionally accepted intervention in a vulnerable population. Its findings will help guide the development of larger, more rigorous trials in the future.

### Conclusion

If our hypothesis is confirmed, moringa supplementation may offer a promising, locally available intervention for mitigating metal-induced oxidative stress during pregnancy. However, conclusions must remain preliminary until supported by completed trial data.

### Future Directions

Future studies should consider RCTs with larger sample sizes and multicenter recruitment to confirm causality. Investigating the dose-response relationship of moringa and including additional biomarkers (eg, GPx and catalase) would offer deeper insights into its antioxidant mechanisms. Longitudinal studies can assess whether reductions in oxidative stress lead to better birth and developmental outcomes. Future research should explore whether reductions in heavy metals and oxidative stress markers translate to improved birth outcomes, such as birth weight, gestational age, or neurodevelopmental status.

### Dissemination Plan

The findings of this study will be disseminated through peer-reviewed journal publications, conference presentations, and policy briefs to local and national health stakeholders. Collaboration with the Makassar City Health Office is planned to integrate results into maternal health programs. Community workshops will also be organized to share findings with participants and local health workers, promoting the evidence-based use of moringa.

## Abbreviations

8-OHdG: 8-hydroxy-2'-deoxyguanosine
Cd: cadmium
Cu: copper
DNA: deoxyribonucleic acid
Fe: iron
Hg: mercury
ICP-MS: inductively coupled plasma mass spectrometry
LBW: low birth weight
MDA: malondialdehyde
Pb: lead
SDG: sustainable development goals
Se: selenium
Zn: zinc

## References

[1] Rai PK, Lee SS, Zhang M, Tsang YF, Kim KH (2019). Heavy metals in food crops: health risks, fate, mechanisms, and management. Environ Int.

[2] Tchounwou PB, Yedjou CG, Patlolla AK, Sutton DJ (2012). Heavy metal toxicity and the environment. Exp Suppl.

[3] World Health Organization Lead poisoning: a persistent public health threat. Regional Office for the Eastern Mediterranean (WHO EMRO).

[4] Neeti K, Prakash T (2013). Effects of heavy metal poisoning during pregnancy. Int Res J Environ Sci.

[5] Rzymski P, Tomczyk K, Rzymski P, Poniedziałek B, Opala T, Wilczak M (2015). Impact of heavy metals on the female reproductive system. Ann Agric Environ Med.

[6] World Health Organization. Lead poisoning and health – Fact sheet.

[7] Shachar BZ, Carmichael SL, Stevenson DK, Shaw GM (2013). Could genetic polymorphisms related to oxidative stress modulate effects of heavy metals for risk of human preterm birth?. Reprod Toxicol.

[8] Koivula MJ, Eeva T (2010). Metal-related oxidative stress in birds. Environ Pollut.

[9] Jomova K, Valko M (2011). Advances in metal-induced oxidative stress and human disease. Toxicology.

[10] Law PP, Holland ML (2019). DNA methylation at the crossroads of gene and environment interactions. Essays Biochem.

[11] Cheng Y, Xie N, Jin P, Wang T (2015). DNA methylation and hydroxymethylation in stem cells. Cell Biochem Funct.

[12] Ehrlich M (2019). DNA hypermethylation in disease: mechanisms and clinical relevance. Epigenetics.

[13] Burris HH, Rifas-Shiman SL, Baccarelli A (2012). Associations of LINE-1 DNA methylation with preterm birth in a prospective cohort study. J Dev Orig Health Dis.

[14] Lee E, Hong S, Kim YD, Lee DI, Eom SY (2024). Evaluating the impact of airborne fine particulate matter and heavy metals on oxidative stress via vitamin supplementation. Toxics.

[15] Alrashed M, Tabassum H, Almuhareb N (2021). Assessment of DNA damage in relation to heavy metal induced oxidative stress in females with recurrent pregnancy loss (RPL). Saudi J Biol Sci.

[16] Killian B, Yuan TH, Tsai CH, Chiu THT, Chen YH, Chan CC (2020). Emission-related heavy metal associated with oxidative stress in children: effect of antioxidant intake. Int J Environ Res Public Health.

[17] Bai Y, Jiang LP, Liu XF (2015). The role of oxidative stress in citreoviridin-induced DNA damage in human liver-derived HepG2 cells. Environ Toxicol.

[18] Qiu C, Hevner K, Abetew D (2013). Mitochondrial DNA copy number and oxidative DNA damage in placental tissues from gestational diabetes and control pregnancies: a pilot study. Clin Lab.

[19] Lean SC, Jones RL, Roberts SA, Heazell AEP (2021). A prospective cohort study providing insights for markers of adverse pregnancy outcome in older mothers. BMC Pregnancy Childbirth.

[20] Holmes VA, McCance DR (2005). Could antioxidant supplementation prevent pre-eclampsia?. Proc Nutr Soc.

[21] Tang Y, Zhang X, Zhang Y (2021). Senescent changes and endoplasmic reticulum stress may be involved in the pathogenesis of missed miscarriage. Front Cell Dev Biol.

[22] Biri A, Bozkurt N, Turp A, Kavutcu M, Himmetoglu O, Durak I (2007). Role of oxidative stress in intrauterine growth restriction. Gynecol Obstet Invest.

[23] Yaman S, Hançerlioğulları N, Tokmak A, Ayhan S, Alışık M, Erel Ö (2019). Impaired serum thiol/disulphide homeostasis may be another explanation for the pathogenesis of missed abortion. Clin Exp Obstet Gynecol.

[24] Zinia SS, Yang KH, Lee EJ (2023). Effects of heavy metal exposure during pregnancy on birth outcomes. Sci Rep.

[25] Tomilola D O (2018). Effect of cadmium on female reproduction and treatment options. Research J of Obstetrics and Gynecology.

[26] Albasher G, Alrajhi R, Alshammry E, Almeer R (2021). Moringa oleifera leaf extract attenuates Pb acetate-induced testicular damage in rats. Comb Chem High Throughput Screen.

[27] Mallya R, Chatterjee PK, Vinodini NA, Chatterjee P, Mithra P (2017). *Moringa oleifera* leaf extract: beneficial effects on cadmium induced toxicities - a review. J Clin Diagn Res.

[28] Brar S, Haugh C, Robertson N (2022). The impact of Moringa oleifera leaf supplementation on human and animal nutrition, growth, and milk production: a systematic review. Phytother Res.

[29] Munir M, Khan I, Shabbab Almutairi N, Hamdan Almutairi A, Khan B, Mehboob N (2025). Effect of moringa leaves powder on body weight, glycemic status, lipid profile, and blood pressure in overweight individuals with hyperlipidemia. Ital J Food Sci.

[30] Derbo ZD, Debelew GT (2024). Maternal fresh moringa leaf consumption and its association with birth weight in southern Ethiopia: a prospective cohort study. Food Sci Nutr.

[31] Ashfaq S, Sajid U, Khan S (2024). Effect of Moringa oleifera leaves powder on hemoglobin level in second-trimester pregnant women of Karachi, Pakistan. Int J endorsing health sci res.

[32] Rotella R, Soriano JM, Llopis-González A, Morales-Suarez-Varela M (2023). The impact of Moringa oleifera supplementation on anemia and other variables during pregnancy and breastfeeding: a narrative review. Nutrients.

[33] Sreelatha S, Padma PR (2009). Antioxidant activity and total phenolic content of Moringa oleifera leaves in two stages of maturity. Plant Foods Hum Nutr.

[34] Asiedu-Gyekye IJ, Frimpong-Manso S, Awortwe C, Antwi DA, Nyarko AK (2014). Micro- and macroelemental composition and safety evaluation of the nutraceutical *Moringa oleifera* leaves. J Toxicol.

[35] Luqman S, Srivastava S, Kumar R, Maurya AK, Chanda D (2012). Experimental assessment of Moringa oleifera leaf and fruit for its antistress, antioxidant, and scavenging potential using in vitro and in vivo assays. Evid Based Complement Alternat Med.

[36] Awodele O, Oreagba IA, Odoma S, da Silva JAT, Osunkalu VO (2012). Toxicological evaluation of the aqueous leaf extract of *Moringa oleifera* Lam. (Moringaceae). J Ethnopharmacol.

[37] Khuzaimah A, Hadju V, As S, Abdullah N, Bahar B, Riu DS (2015). Effect of honey and Moringa oleifera leaf extracts supplementation for preventing DNA damage in passive smoking pregnancy. Int J Sci Basic Appl Res.

[38] Onyeaghala A, Agwu MC, Ogungbemile BD (2024). Effect of supplementation with *Moringa oleifera* on antioxidant and oxidative stress biomarkers of infertile women: a pilot open-label case-control randomized clinical study.

[39] Rahma R, Hadju V, Arsin AA (2023). The effect of moringa leaf extract intervention since preconception period on the prevention of oxidative stress in pregnant women and adverse pregnancy outcomes. Pharmacogn J.

[40] Ali K, Iqbal A, Bukhari SM (2021). Amelioration potential of *Moringa oleifera* extracts against sodium arsenate induced embryotoxicity and genotoxicity in mouse (*Mus musculus*). Braz J Biol.

[41] Velaga MK, Daughtry LK, Jones AC, Yallapragada PR, Rajanna S, Rajanna B (2014). Attenuation of lead-induced oxidative stress in rat brain, liver, kidney and blood of male Wistar rats by *Moringa oleifera* seed powder. J Environ Pathol Toxicol Oncol.

[42] Laksana ASD, Notopuro H, Mustika A (2023). Ameliorative effects of moringa (*Moringa oleifera* Lam.) leaves extract on lead-induced oxidative stress, hepcidin and δ-ALAD levels in rat’s blood. Pharmacogn J.

[43] Puspitasari D, Lestari I, Rahayuningsih CK, Christyaningsih J (2022). The effectiveness of *Moringa oleifera* leaf extract on hepatotoxic case reviewing from cadmium, SGOT and SGPT blood levels in white rats (*Rattus norvegicus*) induced with cadmium (Cd). jteknokes.

[44] Rahayuningsih CK, Agustin R, Idayanti T (2022). Potensi Daun Kelor (*Moringa oleifera*) Terhadap Kadar Kadmium Dan Kreatinin Dalam Darah Sebagai Indikator Kerusakan Fungsi Ginjal Pada Tikus Putih Yang Terpapar Asap Rokok. THE JAMMILT.

[45] Mahmoud K (2021). Appraisal role of watery and acetonic leaves extract of *Moringa oleifera* growing in Egypt against Cd-induced toxicity in male Albino rats. Egypt Acad J Biol Sci F Toxicol Pest Control.

[46] Zn J (2018). Detoxificating effects of *Moringa oleifera* leaf and *Zingiber officinale* root powder on cadmium toxicity in blood and fur of Wistar rats. Open Access J Transl Med Res.

